# Report from the COVID-19 Virtual Summit, *Disaster Experts Speak Out*, March 31, 2020

**DOI:** 10.1017/S1049023X20000552

**Published:** 2020-04-21

**Authors:** James P. Phillips, Luca Ragazzoni, W. Greg Burel, Frederick M. Burkle, Mark Keim

**Affiliations:** 1. Assistant Professor, Department of Emergency Medicine Chief, Section of Disaster and Operational Medicine, The George Washington University School of Medicine and Health Sciences, Washington, DC USA; 2. Assistant Professor and Scientific Coordinator, CRIMEDIM - Research Center in Emergency and Disaster Medicine, Università del Piemonte Orientale, Novara, Italy; 3. President, Hamilton Grace LLC; Fellow, National Academy of Public Administration; Director (Ret.) Strategic National Stockpile, Office of the Assistant Secretary for Preparedness and Response (ASPR), Washington, DC USA; 4. Professor (Ret.), Senior Fellow & Scientist, Harvard Humanitarian Initiative, Harvard University & T.H. Chan School of Public Health, Boston, Massachusetts USA; Senior International Public Policy Scholar, Woodrow Wilson International Center for Scholars, Washington, DC USA; 5. CEO, DisasterDoc, LLC, Lawrenceville, Georgia USA; Adjunct Assistant Professor, Emory University Rollins School of Public Health, Atlanta, Georgia USA; Faculty, Beth Israel Deaconess Medical Center Disaster Medicine Fellowship, Harvard Medical School, Boston, Massachusetts USA

**Keywords:** coronavirus, COVID-19, crisis management, epidemics/pandemics, global public health, public health emergencies

## Abstract

This article captures the webinar narrative on March 31, 2020 of four expert panelists addressing three questions on the current coronavirus disease 2019 (COVID-19) pandemic. Each panelist was selected for their unique personal expertise, ranging from front-line emergency physicians from multiple countries, an international media personality, former director of the US Strategic National Stockpile, and one of the foremost international experts in disaster medicine and public policy. The forum was moderated by one of the most widely recognized disaster medical experts in the world. The four panelists were asked three questions regarding the current pandemic as follows:

1. What do you see as a particular issue of concern during the current pandemic?
2. What do you see as a particular strength during the current pandemic?
3. If you could change one thing about the way that the pandemic response is occurring, what would you change?

## Introduction

This manuscript represents the transcript of a Webinar-Virtual Summit^1^ originally presented on March 31, 2020 to an audience of over 1,000. Many listeners world-wide requested the written transcript. This represents an edited version of the presentations.

The panel was comprised of the following disaster experts from Italy and the United States: (1) Luca Ragazzoni, MD, PhD - emergency and disaster medicine physician and faculty at the Research Center for Emergency and Disaster Medicine in Novara, Italy (very close to the coronavirus disease 2019 [COVID-19] epicenter). (2) James Phillips, MD, FACEP - emergency and disaster medicine physician, Section Chief and Fellowship Director of Disaster and Operational Medicine at The George Washington University School of Medicine and Health Sciences (Washington, DC USA); also, a Medical Analyst on The Cable News Network (CNN; Atlanta, Georgia USA). (3) Greg Burel - public health administrator and former Director of the US Strategic National Stockpile (Office of the Assistant Secretary for Preparedness and Response [ASPR], Washington, DC USA), now President and Principal Consultant at Hamilton Grace LLC consulting group. (4) Frederick “Skip” Burkle Jr., MD - retired professor and now Senior Fellow and Scientist at the Harvard Humanitarian Initiative, T.H. Chan School of Public Health (Boston, Massachusetts USA), and Global Scholar at the Woodrow Wilson International Center for Scholars, Washington, DC USA.

The four panelists were asked three questions regarding the current pandemic as follows:

(1) What do you see as a particular issue of concern during the current pandemic?
(2) What do you see as a particular strength during the current pandemic?
(3) If you could change one thing about the way that the pandemic response is occurring, what would you change?

Panelists’ responses were collected, transcribed, and minimally edited for grammatical flow, not content. This is a manuscript of that transcription. The summit was moderated by Dr. Mark Keim, CEO, DisasterDoc, LLC and adjunct assistant professor, Emory University Rollins School of Public Health (Atlanta, Georgia USA).

## Question #1: What do you see as a particular issue of concern during the current pandemic?

### Response of Luca Ragazzoni, MD, PhD

COVID-19 is now a pandemic that has reached over 100 countries in a matter of weeks. Italy, despite having an advanced health care system, is almost at point break. The situation here is very critical; patients are being treated in hospital corridors, hospital departments have been converted to treat only COVID-19 patients, many doctors and nurses are working outside of their field of expertise, and we are not able to provide the same quality of care as we usually do. Of course, now we know that the intensive care unit (ICU) is one of the most stressed hospital units. However, what is concerning me is that there is not enough attention on the prehospital setting. Primary health care, family doctors, and Emergency Medical Services (EMS) have key roles to alleviate the burden at the hospital before the admission. Additionally, so many doctors and nurses are working outside of their field of expertise that we are not able to provide the same quality of care as we usually do.

We cannot expect that Africa would respond like China, US, and Europe. We cannot ask all the countries to buy ventilators where there are no ICUs and there are a maximum two intensivists in the whole country. We need to differentiate the response and allocate the right resources according to the country.

What we do after we discharge patients from hospitals is really important. In Italy, we need to increase the importance of the role of the prehospital setting. How do we continue care? Unfortunately, currently there is little known about how long the patients need assistance and for how long they remain COVID-19 positive. We need to plan in advance for home care for the patients, or at least create a new health facility to admit patients that are not able to stay at home alone. And of course, what is concerning me apart from the specific issue of the prehospital setting is the global lack of personal protective equipment (PPE). In Italy, we’re having issues trying to find enough PPE for the family physicians, for the prehospital setting, and the hospital staff. Everyday there is a critical lack of PPE, as well as a misunderstanding of the importance of this issue. These are, in my opinion, my concerns currently.

### Response of James Phillips, MD

There are several things that I see as particular concerns right now. PPE is a critical one, not only in the United States where it’s talked about constantly on every major network, but around the world. We talk about Europe and China and the United States ad nauseam, but let’s not forget Africa. Let’s not forget South America. These are places where some of this equipment doesn’t even exist. I saw somewhere today that there’s an African country [Liberia] that has only three ventilators. So, equipment represents a major concern globally, but in particular, PPE is key in the United States where we are rationing it in every hospital. Also, I must emphasize that we are using PPE in a manner that is not consistent with the standard of care. What this means is not that patient treatment is suffering, necessarily, but that provider protection is suffering. I go into work and I put on a mask and I wear it for eight hours. I take it off halfway through, one time, to have an energy drink to keep going. It smells bad after you eat or drink something, and after hours, it becomes hard to breathe through. It makes you very fatigued, and it sometimes makes you breathless to have a conversation with your patient. You can’t understand what anybody is saying in your work area, which has been problematic during critical interventions.

We have seen an outpouring from people who are trying to make PPE for us, but I don’t even know how to begin to advise them, as we cannot wear most of it. I get emails daily from people trying to manufacture cloth masks for us. Some are using 3D printers and Girl Scout Troops to make face shields for us. It’s incredible and heartwarming, and is one of the small bits of hope that we have right now as health care providers, especially when there’s so much disappointing and disheartening news. I do not understand how we can assemble all the power and wealth of the United States to go to the moon or to develop the Manhattan Project, yet we haven’t even considered doing the same thing to manufacture PPE and ventilators. There are a lot of people working hard on this pandemic, but I still think that we haven’t pulled out all the stops. The Defense Production Act is a useful tool, but I think if I were in charge, I would be a little less worried about the economy; in the short term, I’d be more worried about immediately cranking out millions of masks from every textile manufacturer in the United States. We need to raise the curve on PPE production while we try to flatten the curve on the spread of this infection. And if we end up with surplus? That’s what makes us great - because then we can share it with other countries.

While the production process for PPE and ventilators is obviously a concern for us, we are additionally concerned about the ethical decisions that we, as physicians, may be facing as resources run short - when we start talking about rationing. Italy has certainly started that conversation: its Anesthesiology Society published its concerns and guidelines, and that has generated talk here in the United States. I think it was New York University (NYU; New York, USA) hospital whose rationing policy was leaked to the media, raising publicly the potential need for us choosing who gets a ventilator.

This distressing issue is why we, as physicians, need to have our professional societies, medical organizations, and perhaps even the Centers for Disease Control and Prevention (CDC; Atlanta, Georgia USA) weigh in and provide us ethical guidelines so my colleagues and I are not making ad hoc decisions in the heat of the battle. We may be forced to decide which one of three patients needs the last remaining ventilator: a policeman, a mother, or the priest – how do we decide? As it stands now, I alone must choose who gets that ventilator. We need help so that we’re not burdened the rest of our lives by having to make those terrible decisions.

Crisis leadership matters. Once you lie, you lose your credibility and it’s hard to get it back. I turn to my television to watch the President and the Task Force every single day and I am disappointed. Our incident commander, the President, is failing in his ability to communicate the issues at hand. Instead, I find myself simply waiting to hear from Dr. Fauci every day so, that way, I at least hear something I can believe. We need better crisis leadership at the very top, and so I think that one of the lessons we learn from this is the importance that at the highest levels, it cannot be about politics, it’s about crisis leadership.

We also need to address and include all-cause mortality. There’s a lot of people who are going to die from COVID-19 who are never infected with the severe acute respiratory syndrome (SARS) CoV-2 virus. Instead, they will die as a result of the burden on our health care systems. The implications of delayed ambulances and reduced resources could mean dying of a disease or trauma process that, when in non-pandemic times, they would otherwise have been saved. Heroin overdoses that didn’t get narcan in time; severe pediatric allergic reactions that don’t get epinephrine; trauma in general. All those events may happen, and we’re not including those victims right now in the death rate from this pandemic, but they should be counted. These are my biggest concerns that I wish we could address.

### Response of W. Greg Burel

I echo the concerns about the availability of PPE today. I remain concerned about the availability of other material we will need as we move forward in this event. For example, if there is a vaccine that becomes available, or if there are therapeutics that become available, we’re going to need additional needles and syringes - far more than we normally need during the seasonal flu campaign. There are limitations on all products. But, let me go back to the PPE. I agree that the fact that we have limited PPE available for our front-line personnel trying to deal with this problem is just unconscionable. The reason I see behind this dilemma is that the medical supply chain is very fragile, and people don’t really understand that. It’s fragile within all links of the supply chain. What we know about that supply chain is evident for most products, such as chronic disease medications and PPE – from the point of dispensing all the way back to the manufacturer, there is never more than about 30 days of projected need for the entire market available. It’s a very cost-effective system. I think it’s great when you do that for auto parts, I think it’s great when you do that for other materials that are *not* essential to protect and save lives. But when we talk about the medical supply chain, we begin to recognize that problem number one is there is no safety stock. There is no back stock. There is no margin for error in that “just-in-time” delivery of product at any link in the supply chain - all the way from the point-of-care to a distributor or to a manufacturer. That has caused a lot of the problems we have today about getting this vital materiel. Coming out of this on the other side, we’re going to have to really rethink the entire health care supply chain. Do we need to optimize so much for cost or do we need to consider optimizing the costs so that we don’t ever have this kind of a problem in the future? But there are other problems with that supply chain: we are reliant on so many products that are made offshore that are no longer available for manufacturers or have very limited manufacturer availability in the United States. And this is something that many of us have been discussing for years. There’s been Congressional testimony around this problem as well as at the National Academy of Sciences (Washington, DC USA), and many other fora. We’ve got to find a way to get more of this materiel made in the United States. Maybe not all of it, but we’ve got to find a way to protect ourselves better from that supply chain perspective. I think another particular issue of concern goes back to misinformation. There is misinformation and confusion about what the government is doing and not doing, what the Strategic National Stockpile can and can’t do, and a variety of other issues, too. We’ve got to get better about controlling information so that people see the right thing, up first, and credible.

### Response of Frederick M. Burkle, Jr, MD

One, there was and remains a distinct difference in how countries ruled by authoritarian regimes initially managed the early evidence of the pandemic versus democratically ruled countries. And, as one further investigates this, it becomes obvious that in great part, this is a symptom of how both define public health.

In democracies, public health is defined by priorities to improve, protect, and provide services, public health infrastructure, and programs. In authoritarian regimes, public health services, prevention, preparedness, and response are inextricably seen and practiced through political and economic imperatives, always leading to compromises in health and public health security. Currently, the world has the fewest democracies ever recorded, and the United States is currently defined as a “flawed democracy” and is becoming more authoritarian every day.

With this pandemic, I believe it is fair to say that there is a general lack of confidence among health care providers world-wide because, for most, this is their first experience in population-based care. For the most part, they never studied them in medical or nursing schools. In past decades with lower population densities, infectious disease outbreaks, epidemics, and pandemics were rare and driven almost exclusively by large natural disasters, predatory animals, and war. Mine was in 1968 Vietnam with an epidemic of Bubonic plague that not only stopped the war on both sides, it emptied the city and village streets of people for many weeks. While it was the worst plague epidemic of the 20^th^ Century, it was politically kept quiet. Once rare, zoonotic spread (that is between animal reservoirs and human animals) through outbreaks, epidemics, and pandemics now make up 71% or more of new diseases. Globally, they have grown because of massive growth in population numbers and density, which are accelerated further by rapid unsustainable urbanization, biodiversity loss, and climate change; and for the first time, we have an increasing number of vulnerable populations that are suffering from chronic deficiencies in food, water, and energy. We have never seen those numbers before.

And lastly, which I will introduce you to in more depth in discussing the next question: the loss of the World Health Organization (WHO; Geneva, Switzerland) and the International Health Regulations Treaty’s independent leadership established under the United Nations has been devastating; what they were initially organized to do decades ago globally is now absent! They have given their previous leadership role over to political powerhouses across the world because they are monetarily dependent on them. To me, in the long term, this is the most telling issue of concern for us as practitioners and for the future of global public health.

## Question #2: What do you see as a particular strength during the current pandemic?

### Response of Luca Ragazzoni, MD, PhD

This current webinar is an example of what I see as a particular strength during the current pandemic. We are sharing knowledge, we are sharing experience, and we can teach others and explain to others how to prepare in advance. I’ll give you an example. When we had the first cases here in Novara, we started to prepare and to convert the hospital to assist COVID-19 patients. We were treating the first patients in ICU and expecting the waves, and we had a discussion with colleagues everywhere in the world. For example, from Sweden, we had a long talk with Johan von Schreeb - Karolinska Institutet (Solna, Sweden), and health officers there and according to our suggestions, they prepared in advance. Few days ago, I received a message from Johan saying, “Thanks a lot Luca and professor Della Corte to help us in preparing ourselves and to increase the ICU capacity.” And, we had other webinars like this one to help others. I think to share knowledge is a real strength right now.

And, of course, the science: we need to remember everyone, disaster medicine is a science and we need to follow the sciences that are now available in the literature. And, in my opinion, now there is real added value for everyone. And, of course, I have to echo what James said: the willingness of the health care professionals to respond is amazing. Right now, here in Italy, it is spectacular: we had a call for physicians, for nurses to help in hospitals. We called 300 physicians and nurses and we received 9,000 responses. The willingness to help is unbelievable.

### Response of James Phillips, MD

I have to start with the front-line providers - the doctors and nurses at the local level are stepping up and showing their courage. You know as we say in the disaster medicine world, all disasters are local - and while I emphasize the fact that we’re lacking in leadership at the federal level, the good news is locally, that’s not the case. I know that every hospital is different. There’s nothing even remotely one-size-fits-all about this pandemic: there are differences between academic hospitals, non-academic hospitals, and critical access hospitals. But where I work, at George Washington University Hospital, we have a robust disaster medicine program that was in place long before I joined, a faculty team including Anthony McIntyre, Bruno Petinaux, Dan Hanfling, and Joe Barbera - so our hospital is very well-versed in preparing for these crises. I’m able to watch our emergency manager, our local leader, provide great leadership getting our hospital prepared both behind the scenes and in public. I’m able to interact with our nurses and technicians both in the emergency department and the ICU who are all preparing. However, as is being shown in the media, we see courage knowing that many have been told to wear PPE in a way that is not consistent with the standard of care, yet they do not hesitate to go in to treat the most ill of patients. But it is taking toll on us. I received an email a few minutes ago saying that we need coverage for tonight at one of our hospitals because one of my colleagues is now sick with COVID-19 symptoms.

We are just at the tip of what we’re going to see here in Washington DC, evident when we see the incredible evidence of what’s taking place in New York. But I am encouraged, witnessing planeloads full of medical workers flying into New York to volunteer to work. It’s absolutely incredible. I also appreciate Governor Cuomo displaying what I think is very good crisis leadership, and this will be proved true when we look back through the long list of after-action reports.

Following this pandemic, I think one of the things we’re going to see as a silver lining is the expertise provided through telemedicine. I think that the world will never be the same in relation to the Telehealth system, allowing doctors to provide care remotely and in a more efficient manner – and now without a financial loss, which is critical. Also, reducing associated regulations has been key.

### Response of W. Greg Burel

As the former Director of the Strategic National Stockpile, I guess somewhat selfishly that the first thing I see that we do have is the Strategic National Stockpile in the United States. While currently there’s a lot of criticism that it doesn’t have enough of everything, the Strategic National Stockpile is the most-ready of any global stockpile that we’re aware of. It’s one of the crown jewels in our preparedness system. No, it’s not perfect. But every other country looks at us and asks us what we do and how to create their own. However, admittedly the problem that is facing us right now is that the Strategic National Stockpile has been chronically underfunded for years and we hope we see that improve. I think another particular strength that we’re seeing, and it’s been alluded to before, is the way that people all across the United States, and in other countries as well, are coming together to try to help solve some of these problems. There are limitations to what you can do, but the fact that people are willing to step up and try is a major strength.

I think we’re seeing more people trying to abide by the “remain in place and isolate yourself” policy to a certain extent than what we might have thought we would. We’ve got people doing some crazy things out there, but you’re always going to have that. A final strength I see right now is the decision that the Federal Emergency Management Agency (FEMA; Washington, DC USA) can respond to biological events. In 2009, when we responded to H1N1, they didn’t have that capability. So I think FEMA leading the National Response Framework and bringing together the whole of government to try to address this problem puts us in a better position than we would have been. Otherwise, we must remember it’s their first experience. There are going to be hiccups and they’re going to be things that we need to go back and look at, as many of us know. As these after-action hot washes go, we’ll beat ourselves to death for several years over it. But I think that this is good. I think it’s brought new capability to a table that might not have been there before. This whole of government response I think is going to be important to us moving ahead.

### Response of Frederick M. Burkle, Jr., MD

First, I guess, would be to see the health care providers speaking up, first against China’s delays and inexcusable political decisions that violate public health dictums, and similar concerns raised by health care providers in other countries. Remember, the virus was known as early as last October, not the December date the Chinese first claimed. The delay was inexcusable!

Second, clearly, the manner in which health care providers volunteered and had to quickly learn the nuances of population-based care. My one published study on triage categories in pandemics was the first, and only triage structure, that had a category for the “non-survivable.” I believe Italy was the first compelled in population-based care to use this category. This has been listed as the most difficult of decisions, and the first ever except in war, that health care providers have had to make.

Lastly, I would offer that the pandemic and all its management deficiencies gives global public health some needed recognition. It cannot turn back, but the changes that must take place globally will be resisted in draconian ways by authoritarian regimes. This pandemic will, of course, challenge “globalization,” which is totally failing because it only focused on economics - never public health - and it should fail. But we must ensure that this pandemic will strengthen what we now know as global public health.

## Question #3: If you could change one thing about the way that the pandemic response is occurring, what would you change?

### Response of Luca Ragazzoni, MD, PhD

I would say three main aspects: stronger containment measures, stronger coordination between health sectors, and more just-in-time training. No health care system can sustain an uncontrolled outbreak, and stronger containment measures are now the only realistic option to avoid the total collapse of any health care system. All governments must act now implementing traditional public health response tactics (isolation, quarantine, social distancing, community containment, and aggressive lockdown). Also, all people must know the basics of infection prevention and control measures, and all precautionary measures aimed at avoiding the collapse of your health care system and an unnecessary loss of lives.

Also, from my perspective in Italy, we need more and stronger coordination between the different health sectors, from the prehospital to the hospital, more coordination between family doctors, between EMS and hospitals to contain the admission of patients in-hospital and to continue the care at home after the discharge. And I would like to spend a little bit more time on training since it’s my field of expertise. I would say we need more training, more just-in-time training, not only on donning and doffing PPE, so I echo what Skip said: we are lacking of education in training in the medical schools, in the nurses’ schools, at any level. So, right now, we need to act and to improve what we didn’t implement before. We need to increase the knowledge about triage, about ethical dilemmas in this difficult situation. It’s not just about donning and doffing PPE. And also, on basic principles of infection prevention and control. Again, it’s not only because you’re wearing a mask that you’re protected. So, we need to teach everyone. We need to educate health professionals on how to protect themselves and then also on how to react and to respond to difficult situations. For example, again, how to expand the hospital, how to triage patients at the admission, and how to triage patients to decide which one needs ICU, needs intubation, and which one not. I think these are principles that are extremely important right now.

### Response of James Phillips, MD

I mentioned PPE before, and you know on an individual level as a physician on the front lines, that is our major need. But to think in terms of population health and a crisis management framework: we can change the PPE issue and we can change ventilators. Those are things that we can improve and improve quickly. But we talked about crisis leadership - and that’s not something we are able to change right now. We are getting messaging from the President that is very difficult to hear as a front-line health care provider. I don’t feel like he has my back and that’s unfortunate.

This is not a political issue: the coronavirus is neither a Democrat nor Republican, and neither am I. But we look to the President as the incident commander in a time of crisis like this. The President has a choice of two ways to lead. One can either lead by inspiration and rally the troops into battle, or you can confuse them via mixed messaging. Even accusing us of stealing masks and selling them out the back door, like what happened recently. It is completely demoralizing. We have to rely on our governors and our local experts. We know we need containment, and that the President has the power to enact national containment, but won’t. Our Governors are going to finally have to reach a decisional critical mass before the President would ever decide to make a national decree, if he will at all. As the number of states where we have lockdown procedures grows, like Maryland and my home of Virginia declared yesterday, only then will we possibly see a federal change.

More personally, crisis communication is one of the things that inspired me into disaster medicine. I was 16 when the Oklahoma City bombing occurred (1995; Oklahoma USA) - it rattled my window; I heard the boom. My father was one of the first firefighters in the building, going straight into the nursery minutes after detonation. Since that time, I knew this is what I was supposed to do as a career. I recall that every night we would come home and there on TV was the excellent Oklahoma City Fire Department Public Information Officer (PIO) named Jon Hanson. Jon was on every single night at the same time telling us what they knew, what they didn’t know, and how they were going to find out. When he was on the air, we all felt that things were going to be okay, because the people in charge were honest and informed. That’s what America needs now. And, because we’re Americans, the world looks to us. I don’t mean to be an American exceptionalist, but I think we have missed a critical opportunity to provide global leadership at that highest level. So, if I could change one thing, it would be the quality of our national leadership.

### Response of W. Greg Burel

I would say crisis leadership again, and I look at that from a couple of different lenses. The first is, we learned a long time ago that you must be first and you must be credible. And I’m afraid that we’ve missed that boat to a certain extent. I think back to 2009 and H1N1. We had a late-night phone call when we were beginning to see these cases and we began to understand what it might mean to the United States. The CDC Director was on camera the next morning, first thing, talking about what we knew, talking credibly about what needed to be our concerns. And we didn’t see that here, and that bothers me. Another issue we need to think about from a crisis leadership perspective is beyond just whether we’re going to send these things out or are we going to award some contracts or whatever. We need to be taking better control at the Federal level of these supply chain problems. I was horrified to hear the comment that states should just go out and try to get their own things, all at the same time the federal government is trying to do the same. We’ve set up a competitive environment between the states and the federal government. There is no room for that kind of competitiveness when we all need to be pulling together to respond to this event nation-wide. I have never been a strong proponent of rapidly running out the door and placing orders under the Defense Production Act because that puts the federal government first in line. But in this particular case, with some of these particular commodities, like PPE, that it is absolutely harming our response. I believe that the government must order under the Defense Production Act – put itself first in line, bring in other capabilities to bear that are not normally engaged in this production field, and then make those allocations, as difficult as that might be, across the country. Versus having every health care facility in every state and every locality and the federal government all competing with each other for these scarce products. It’s creating an issue across this entire supply chain, including placing equipment that is not needed. As of this writing, supplies are going where it is not needed and not going to where it is desperately needed now. So back to the crisis leadership, whereas I wanted to give you something different, I think this is the most important issue we need to see changing and need to see immediate improvement. I trust Dr. Tony Fauci.

### Response of Frederick M. Burkle, Jr., MD

First, we need to change the medical and public health curriculum globally to include zoonotic diseases, or those that are spread from reservoir animals to human animals. Currently, this has not been a priority in education and health delivery. Medical efforts must not be directed at response phase alone, but across the entire disaster cycle: prevention, preparedness, response, recovery, and rehabilitation. Health must take ownership. This applies to all health populations, as well as being supported by the societies and the governments they live in!

The WHO and its International Health Regulation Treaty, organized to manage population-based diseases such as Influenza, SARS, H1N1, MERS, HIV, and Ebola, have failed to meet population-based expectations. In part, this is due to influence from powerful political donors which the WHO is totally dependent on for their existence, and which has become most painfully evident in the current COVID-19 pandemic. The WHO must exist solely as a treaty-based organization sanctioned and totally funded by the United Nations and all its members. It cannot be dependent on outside financial assistance to do its work.

The global community can no longer tolerate an ineffectual and passive international response system, nor tolerate the self-serving political interference that authoritarian regimes and others have exercised over the WHO and expect to do in the future! In a highly integrated globalized world, both the WHO and its Treaty have the potential to become one of the most effective mechanisms for crisis response and risk reduction world-wide. Practitioners and health decision-makers must break their silence and advocate for a stronger treaty, a return of the WHO’s singular global authority, and support a highly coordinated population-based management. As Sir William Osler, who was the father of zoonotic diseases, recognized his concept of “one medicine, one health,” first written in the early 1900’s, defines what global public health is striving for today.

Public health and public health infrastructure and systems in developing countries must be seen as strategic and security issues that deserve international public health resource monitoring. Again, this must cover the entire disaster cycle from prevention, preparedness, response, recovery, and rehabilitation. Also, all six WHO Regional Offices must have similar multidisciplinary professional assets in support of zoonotic sciences which, of course, would be further resourced from WHO global assets during any epidemic or pandemic. The goal is to identify and begin the containment process as early as possible.

I must say that America is going to do poorly. The 50 states are performing as 50 different countries - it is insane! The individual states’ Departments of Health are all run by political appointees and differ greatly in capability, capacity, and containment decisions. Some departments are excellent models of efficiency while others do not even have a department building to call their own.

Lastly, collectively, we must begin now to think and plan for a new generation in every country of the credentials of health care managers and scientists who are trained across the entire disaster cycle to anticipate and manage population-based crises. Epidemics and pandemics are not the only entities that have the potential to devastate societies. Nuclear war, climate change, and biodiversity loss alone can lead to global devastation. However, I am aware that my Italian colleagues, before this pandemic occurred, have seriously addressed and requested funding for a PhD-level training program for professional health care managers and scientists within the European Union. This must be supported to fuel the expertise of a future WHO and International Health Regulation globally as well as in individual countries! There is no excuse!

## Summary

The three questions posed to the expert panel, moderated and summarized by Dr. Keim in real-time, are summarized here in toto.

Particular concerns the members of the panel expressed focused on the scarcity of PPE and the fragility of the supply chain for our health care system, as well as the Strategic National Stockpile. Two members felt the Defense Production Act was being under-utilized in regard to emergency manufacturing of resources, and there is concern this could force physicians to make life or death resource rationing decisions without guidance. The Italian system is currently stressed in terms of space, staff, and equipment, and more focus should be placed on the outside of the hospital setting, including EMS, primary care, and post-discharge care. Disaster response is certainly made more complicated by the type of government philosophy and attitude in different countries. Deficiencies in medical education in global health are also being seen.

The second question prompted answers that uniformly praised the response of the individual health care worker and the general public response across Western countries. Similarly, knowledge sharing between countries has been a strength. The Strategic National Stockpile has been used to address shortages and has saved lives; however, its limitations have been now made apparent. FEMA has been granted the authority to respond to pandemic events, which is new, and is also seen as a positive. Global Public Health is also having its moment in the sun and showing its value.

The final question generated enthusiastic answers in regard to failures in leadership at the highest levels. It was stated or implied that the President and the WHO have extremely valuable roles during times of crisis such as this, and it was universally believed among the American panelists that there has been a failure by both in regard to crisis leadership and communication. Better pandemic response education and communication within hospitals was also highlighted.
